# PRICHAIN: A Partially Decentralized Implementation of UbiPri Middleware Using Blockchain

**DOI:** 10.3390/s19204483

**Published:** 2019-10-16

**Authors:** Iago Sestrem Ochôa, Luis Augusto Silva, Gabriel de Mello, Bruno Alves da Silva, Juan Francisco de Paz, Gabriel Villarrubia González, Nuno M. Garcia, Valderi Reis Quietinho Leithardt

**Affiliations:** 1 Laboratory of Embedded and Distributed Systems-LEDS, University of Vale do Itajaí, Itajaí-SC 88302-901, Brazil; luis.silva@edu.univali.br (L.A.S.); gabrieldemello@edu.univali.br (G.d.M.); silvabruno@edu.univali.br (B.A.d.S.); valderi.leithardt@ubi.pt (V.R.Q.L.); 2 Departamento de Informática e Redes de Computadores, Instituto Federal Catarinense (IFC), Brusque 88354-300, Brazil; 3 Expert Systems and Applications Lab, Faculty of Science, University of Salamanca, Plaza de los Caídos s/n, 37008 Salamanca, Spain; fcofds@usal.es (J.F.d.P.); gvg@usal.es (G.V.G.); 4 Departamento de Informática, Universidade da Beira Interior, 6201-001 Covilhã, Portugal; ngarcia@di.ubi.pt; 5 Instituto de Telecomunicações, Universidade da Beira Interior, 6201-001 Covilhã, Portugal; 6 COPELABS, Universidade Lusófona de Humanidades e Tecnologias, 1749-024 Lisboa, Portugal

**Keywords:** privacy, blockchain, ethereum

## Abstract

With the popularization of the Internet-of-Things, various applications have emerged to make life easier. These applications generate a large amount of user data. Analyzing the data obtained from these applications, one can infer personal information about each user. Considering this, it is clear that ensuring privacy in this type of application is essential. To guarantee privacy various solutions exist, one of them is UbiPri middleware. This paper presents a decentralized implementation of UbiPri middleware using the Ethereum blockchain. Smart contracts were used in conjunction with a communication gateway and a distributed storage service to ensure users privacy. The results obtained show that the implementation of this work ensures privacy at different levels, data storage security, and performance regarding scalability in the Internet of Things environments.

## 1. Introduction

In recent years, the term IoT has become popular all over the world, inciting the attention of researchers from different areas. One of IoT main goals is to make the Internet even more immersive. Also, by facilitating access to a wide range of devices such as appliances, surveillance cameras, monitoring sensors, actuators, vehicles, and others; the Internet becomes more interactive. According to [1], by 2020, 50 billion devices will be connected to the Internet. Thus, IoT will enable the development of different applications that make use of the data generated by such devices to provide new services to citizens, businesses, and governments. However, connecting these devices still be one of IoT biggest challenges. According to [2], the use of centralized architectures still popular today, with authentication, authorization, and connection of devices; This model is sufficient for the current development of IoT, however, with their growth, these systems will need more investments, and entire systems could be damaged if the centralized point of the architecture becomes unavailable.

Considering the centralized architecture model, in addition to scalability issues such as IoT growth, there are still security, privacy, and failure issues. These problems occur due to centralized architectures run services on a single point of the network. The moment that the single point fails, the entire system is compromised. Nowadays, there are various proposals for centralized architectures for IoT environments. The work proposed in [3] describes a software-defined device-based centralized architecture for IoT environments. In the work presented in [4], the authors describe a centralized architecture using Fog-to-Cloudlet-to-Cloud Data Management in Smart Cities. In [5], the authors propose a centralized clustering-based architecture for communication between smart homes. However, as mentioned earlier, centralized architectures are vulnerable to different issues that can compromise the system. The privacy leak problem can be considered one of the most dangerous in this type of environment.

With a massive amount of data generated in IoT environments, different privacies may exist. Four main concepts define privacy, they are information privacy, body privacy, the privacy of communication, and territorial privacy. The work proposed by [6] shows an architecture that manipulates privacy at different levels. This approach is ideal for IoT environments, given a large number of devices connected in different environments by multiple users. However, addressing privacy at different levels using centralized architectures becomes problematic due to the unique points of failure in this type of architecture. This approach requires the use of a decentralized and robust technology that can solve the privacy problem completely, thus ensuring the proper functioning of IoT applications.

In 2008, Nakamoto described the blockchain concept through the Bitcoin cryptocurrency [7]. Blockchain is a decentralized technology that ensures the integrity of transactions performed on the platform without the need for validation by a third party. The blocks store the transactions done and link to each other through a hash function. A consensus algorithm, also called mining process, calculates the hash of each block. The mining process varies for each blockchain. Altering a block maliciously will compromise all other existing blocks in the chain, then all blocks need to be re-mined to be valid, making the process of hacking a blockchain unfeasible. Other applications than cryptocurrency can benefit from the blockchain technology. Over the years, different blockchains came up with different proposals for different scenarios. One of the most promising blockchains is the Ethereum network.

Vitalik Buterin proposed the Ethereum platform in 2013 [8]. Ethereum platform is a decentralized network able to execute smart contracts. These are programmed scripts that work as programmed, without the possibility of fraud, censorship, or alteration through third parties. Due to this functionality, this platform has proved to be revolutionary for the development of decentralized applications. Diverse decentralized applications are proposed using the Ethereum platform, as examples are marketplace [9], e-voting [10], smart grid [11], social networks [12], smart home [13], e-health [14], fake news detection [15], and others. Considering this, it is clear that the Ethereum platform is viable for the development of IoT applications of various types due to its functionalities.

Considering this context, we propose an architecture that uses the Ethereum platform for privacy management in IoT environments. In our architecture, we use smart contracts to manage privacy at different levels for different profiles. We chose to use a gateway to manage device-user communication, considering the scalability of the network. The scenario chosen for testing was an educational environment in different situations. We developed a decentralized mobile application to prove the efficiency of the proposed architecture.

This paper is structured as follows. Section 2 presents the background necessary for understanding the proposed architecture. In Section 3, an analysis of related work is done, considering the privacy management in IoT environments. In Section 4, we describe the proposed architecture. Section 5 describes the tests done and the results obtained. Finally, Section 6 presents the conclusions obtained from the development of this work and the suggestions for future work.

## 2. Background

### 2.1. Blockchain

**Figure 1:**
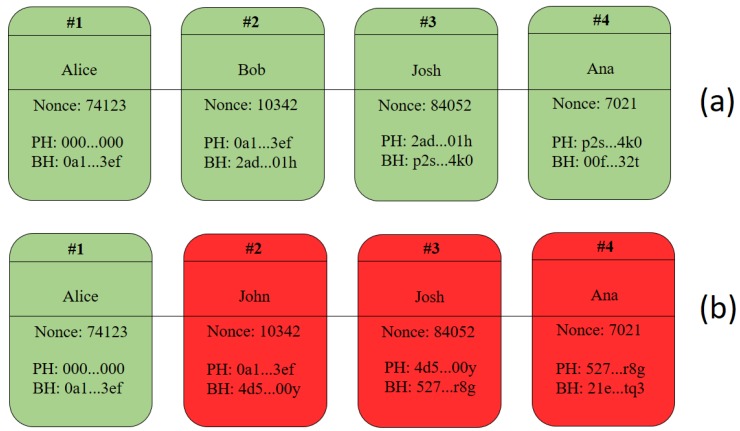
(**a**) Original blockchain and (**b**) Changed blockchain.

Blockchain technology is a decentralized platform for transaction storage. Inside each block information is stored regarding the transactions performed, the block generation time, the current block hash, the previous block hash, and the nonce value. The nonce value is used to find the correct hash of a block. This process may vary for each blockchain, but they all have the same purpose—to indicate the correct hash value. Figure 1 illustrates the process described. In (a) a simple blockchain is built and validated. In (b) the second block is altered, compromising the entire blockchain, requiring the mining of all blocks after the second one to validate the entire ledger.

The mining process consists of a consensus algorithm. This kind of algorithm may change in each blockchain, on Bitcoin and Ethereum blockchains use Proof-of-Work (PoW) algorithm, which consists in solving a cryptographic problem by brute force. On Hyperledger blockchain is used the Byzantine Fault Tolerance consensus algorithm [16]. The use of different consensus algorithms in blockchains happens due to scalability, security, and efficiency issues. The Ethereum blockchain processes approximately 15 transactions per second using the PoW algorithm. As far as security is concerned, to be able to change the Ethereum blockchain, one of the nodes on the network needs 25 % of the total processing power of the network to do it. In terms of efficiency, the Ethereum platform consumes more electricity than the countries of Myanmar and Tajikistan [17].

Aiming to solve scalability issues, private blockchains are becoming increasingly popular. This  type of blockchain, in terms of performance and scalability, is more efficient than public blockchains. This efficiency happens because transactions do not necessarily have fees to be processed. In public blockchains, the high number of nodes and the fees charged for processing each transaction infer scalability and performance issues. In private blockchains, characteristics are defined individually like the number and permissions of each node. Another type of blockchain used is the consortium, this type of blockchain allows many people to participate in the blockchain, but only a few nodes participate in the mining process [18,19].

Regarding the storage of generated data, this classification has two kinds. When the blockchain stores all the generated data, we call this the on-chain storage process. This type of storage is less favorable in terms of performance, cost, and scalability. However, even though it is the least favorable type of storage in the issues mentioned above, it still respects the blockchain operating principles. When external storage services store the data generated in the blockchain, we call this the off-chain storage process. In performance, cost, and scalability issues, this solution may be better. However, this process compromises the transparency and immutability of the data. Today, most decentralized applications are developed using off-chain storage services [20].

With the evolution of technology, over the years, different blockchains have appeared trying to solve existing daily problems. HyperLedger Fabric is a platform of the Linux Foundation for the development of industry-specific permissioned blockchains [21]. Focused on IoT environments, the IOTA blockchain was developed to address existing problems in another blockchains [22]. Ripple is a payment protocol that uses blockchain specifically for financial transactions [23]. However, one of the most promising platforms is the Ethereum network, which guarantees the complete user control over their applications and information [24].

### 2.2. Ethereum Blockchain

In 2014, the Ethereum platform was introduced to the world by Vitalik Buterin [8]. Ethereum is not only a cryptocurrency but is also a programming language running on a blockchain. This platform allows users to create smart contracts and transfer cryptocurrencies to other users by validating these contracts. According to [25], a smart contract is a self-checking, self-executing, and anti-fraud computer program. Smart contracts aim to facilitate negotiation between strangers by eliminating the need for a third party to audit the negotiation. Existing applications for smart contracts are diverse, such as breach prevention, financial services, credit application, among others. The mining process validates the contracts and information contained in the blockchain.

**Figure 2:**
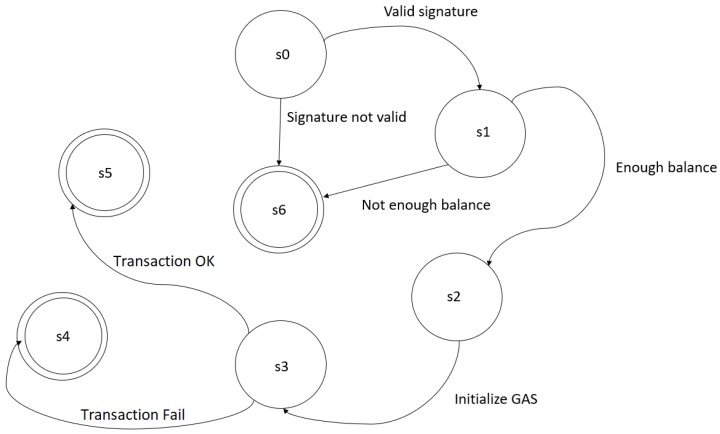
Ethereum state machine.

The term transaction is used in the Ethereum platform to relate to data packets that store messages between users. The transactions contain the message receiver, a signature identifying the sender, the amount of Ether, and the data to send. Transactions also contain two values called gas price (value paid in Ether by a unit of gas spent) and gas limit (maximum gas amount to use) [26]. Each  operation performed on the Ethereum platform has a cost called gas [8]. The gas cost varies for each operation. For  example, storing a contract on the Ethereum platform costs 32,000 GAS, while storing a 256-bit value has a cost of 20,000 GAS. Figure 2 illustrates the operation of a transaction on the Ethereum network.

State s0 corresponds to transaction validity check. State s1 calculates the fee to be charged for the transaction and subtracts the fee from the sender’s account. The s2 state is responsible for initializing gas. The s3 state transfers the transaction value from the sender to the recipient. The state s4 corresponds to a failed transaction due to lack of currency or gas from the sender, if this happens all states are reversed except the payment of fees. State s5 represents a transaction that occurred correctly, so fees for the amount of gas leftover are refunded to the sender, fees for gas consumed are sent to the forger. State s6 corresponds to errors that can occur in states s0 and s1.

Network users set the gas price. At the time this work was developed, the gas price was 20 GWei. The gas price varies according to the operation of the network. The value shown above is the default gas price, meaning that the user who pays this price for the gas used will have their transaction confirmed on the Ethereum platform within 30 min. If the user wants to the transaction be confirmed faster on the blockchain, they must pay a value of 80 GWei perused gas, having their transaction confirmed in less than 2 min. Information regarding the Ethereum platform values can be obtained from [27]. It is necessary to mention that the limit of transactions per block in the Ethereum platform is defined by the value corresponding to the maximum gas that can be executed in a block. Users who mine the Ethereum network blocks set this value. At the time of this work, the gas value per block was equal to 8,000,000 [27]. Considering the popularization of the Ethereum platform over the years, this technology has been used to develop and optimize different applications.

### 2.3. UbiPri Middleware

**Figure 3:**
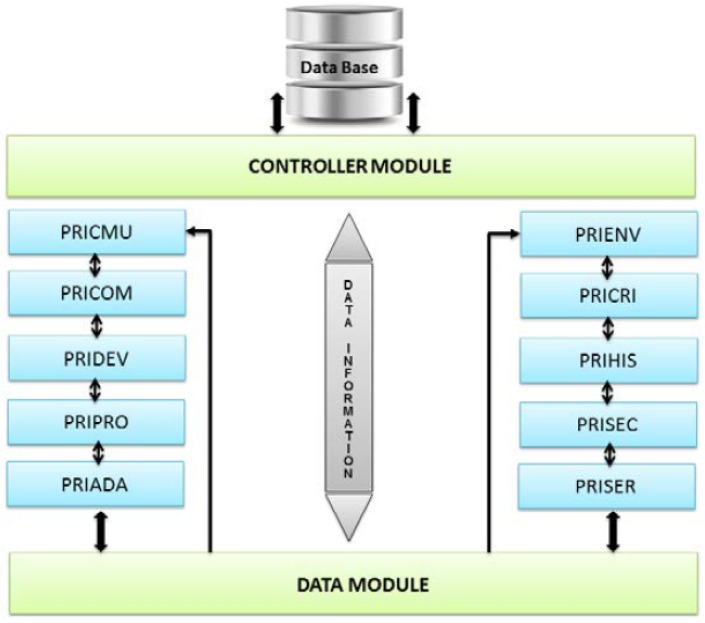
UbiPri Middleware [28].

UbiPri [28] middleware is an architecture defined to allow a given device to be geared to meet the needs of a user, or the environment as a whole, adapting to the environment and its project infrastructure, depending on device limitations and the need for each environment. To ensure its functionality, different modules divide the architecture, each with its management and control function for a given application scenario. Figure 3 illustrates the UbiPri privacy manager model.

As illustrated in Figure 3, UbiPri has in its architecture, a database responsible for storing and defining rules for each user, device, or communication in the ubiquitous environment. The Controller Module has the function of receiving access requests and controlling the Data Base. The Data Module processes all variables and parameters received from other modules. This module also has the function of receiving various data and handling them, generating a single output. The PRICMU, PRICOM, and PRIDEV modules manage and control the privacy of user information, communications, and devices, respectively. The PRIPRO module performs control transactions related to user profile management. PRIADA manages and controls adaptation, responsible for handling information related to software and hardware adaptation in the ubiquitous environment. The PRIENV module has the function of receiving the register of attributes related to the environment. PRICRI has the rules and criteria definitions of the environment. The PRIHIS module stores and manage information related to a user history, environment, and devices. PRISEC is responsible for user and environmental security. Finally, the PRISER module manages environment services, handling service availability information.

**Figure 4:**
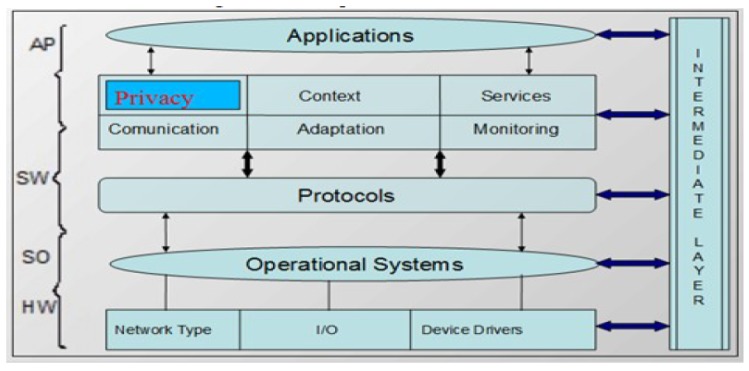
UbiPri Architecture [29].

The UbiPri privacy manager model connects all modules of its architecture, but the modules work independently. A module does not depend directly on another module to perform its functionality. Thus, only the database access is enough for each module to be able to perform its function. Considering the operating standard of UbiPri, it is possible to have several environments with different rules and definitions. The same user can also use different environments, each with their privacy criteria. Four interconnected layers denote the UbiPri architecture encompassing the features and requirements required for controlling wireless sensor networks in ubiquitous environments. The  layers of architecture are hardware, operational system, software, application, and brokering layer. Figure 4 illustrates the middleware architecture.

The hardware layer consists of the network module that is used to connect the platform to the pervasive network. The I/O module is used for communication and interface for users, environments, and devices. The Device Drivers module acts as a trigger for connecting the physical devices registered in the system. The operating systems layer aims to address the operational system functionalities for the embedded system, assisted by the Device Drivers module that manages the physical layer components and sends the information to the Operating System sublayer, which is responsible for managing the application tasks that runs on the device and determines the services it provides, and addresses operating system limitations. The software layer is a set of components needed to assist the device integration and treatment with the ubiquitous network and to provide the services and other necessary functions that make up the middleware in the architecture. This layer is made up of different modules that help in communicating and addressing device, user, and environment preferences. The  application layer is a module that has fragments of the applications that run in the environment. It will perform all the necessary configurations for any application to work in the ubiquitous environment. However, the UbiPri privacy manager is implemented centrally, where a failure could lead to the unavailability of the services offered. Decentralizing this middleware even partially can offer several advantages for using this architecture.

## 3. Related Work

This section shows the related work identified. In the analysis, we attempt to identify in the works listed, how each of the authors treated the privacy preferences of the user, environment, and device. We studied how each of the references dealt with the use of blockchains for scalability and network performance. Also, we observed how the authors addressed the storage of data acquired in each application. At the end of this section, we make a comparison between the identified related works and the proposed architecture in our work, trying to present the advantages of our work about the architecture proposed by other authors.

The work presented in [30] shows the idea of using gateways combined with smart contracts for privacy management in IoT environments. The architecture presented addresses user and device privacy, this happens because the user has privacy preferences registered in one smart contract, and the device preferences are registered in another smart contract. Device-user communication is done through a gateway that is also responsible for executing the blockchain privacy preferences register. For the development of the tests, the authors used a private blockchain built in the Ethereum network. The main critique of this work concerns the registration of privacy preferences in the blockchain, the gateway is used to perform this role, but any failure or malicious access to the gateway could alter existing privacy preferences or register malicious devices in the blockchain network.

In [31], the authors describe a blockchain architecture directed at handling device information and registration in IoT environments. The privacy approached at work refers to the user, who can register through smart contracts their devices, access policies, transactions with other devices, and other features. The authors illustrate an utterly decentralized architecture where, according to them, there is no need for hardware to assist in device-user communication. To be able to exercise communication in a completely decentralized way, the authors report using the Proof-of-Authority consensus algorithm. Data storage is done off-chain through the IPFS protocol. The blockchain used in the tests was the Ethereum blockchain. The main critique regarding this work refers to the use of the Proof-of-Authority consensus algorithm, which is valid only in implementations in which there is a processing node. The  authors use more than one processing node in their architecture. The validation of the implementation is not confirmed since no tests are presenting the algorithm.

The work of [32] proposes a blockchain architecture for IoT environments focused on off-chain data storage using the IPFS protocol. The authors define device privacy through smart contracts that store information such as the cryptographic keys of each device, the devices that are allowed to communicate with the blockchain, and others. This structure ensures device privacy, as a specific environment can have a blockchain (sidechain concept), so each can have different privacy rules and policies. In the tests was used a consortium blockchain built in the Ethereum network. In this work, the negative aspect observed was the number of transactions performed per minute on the blockchain, the authors report the use of 97% of the validation node processing power to reach 300 transactions per minute on the built blockchain for the tests.

The work presented in [33] consists of using blockchain in smart homes, focusing on the privacy of the devices in this type of environment. The privacy addressed at work regards about devices, where through different smart contracts (access control, decision making, and registration) the privacy preferences of each device are controlled and set. In each smart home, a gateway is used to communicate with the blockchain. According to the authors, IoT devices do not have the computational power to communicate directly with the blockchain, so it is necessary to use a gateway to perform this communication. The authors developed the tests on a private blockchain built on the Ethereum network, storing data off-chain through a cloud service. The main critique of this work is that the results obtained show only the functionality tests of smart contracts, disregarding the gateway performance.

In [34], the authors aim to ensure privacy about the IoT data, for which the authors propose a framework using blockchain. In this paper, the privacy of the devices is addressed using smart contracts. Three different contracts (privacy setting, owner, and privacy policy subscription) are used to address the privacy mentioned. The gateway is used to communicate devices with the blockchain. The authors decided to use a gateway because of the performance characteristics of this hardware over IoT devices. The work uses two blockchains, one public and one private, responsible for controlling transactions between different devices in different locations and allowing the user to control their own devices, respectively. The Ethereum blockchain was used to implement the proposed framework. Data  generated by IoT devices is stored off-chain. The main critique regarding this work is the cryptographic key registration; the gateway is responsible for doing this process. If an attacker has access to it, can generate keys for devices that should not belong to the network.

The work of [35] proposes a decentralized architecture focused on IoT environments using smart contracts to ensure autonomy on device communication. The work addresses device privacy, where a smart contract is generated for each device, allowing to define different functions for each device registered in the network (here it is mentioned that each device has a contract with unique functions, being different contracts for each device due to its characteristics). The proposed architecture uses no gateway. In the tests made, the Ethereum blockchain was used. The data storage is done off-chain through a clouding service. The main critique about this paper regards to network performance, considering that there will be a large number of transactions, it is not possible to identify in the authors work, how blockchain can handle a large number of transaction requests between connected devices.

The work presented in [36] aims to transfer the right of access to devices in a decentralized way. The authors propose the use of Tangle technology, present in IOTA blockchain. The privacy addressed at work concerns about the user, where the user sets his privacy preferences and distributes the privacy preference to another user through a token. The user will only receive the token if its features meet the privacy preferences of the token owner. IOTA blockchain offers performance and scalability advantages, so no gateway was used. The main critique regarding this work is privacy issues. The work does not discuss in detail how the privacy policies of users are stored. Compared to other works, we could not identify how that same architecture could treat other types of privacy (e.g., environment and device).

The work of [37] proposes privacy protection through the use of different blockchains to prevent transaction tracking attacks. The privacy mentioned by the authors regards to user’s privacy, which, through their shared information, may allow someone to find out their location by tracking the transactions stored in the blockchain. Different private blockchains are used to redistribute the transaction made by the user across different networks. The public blockchain is used to initiate the transaction process between two network users. The authors do not use gateways in their work. The main critique about this work is the cost of implementing the solution proposed by the authors for other scenarios since different blockchains are present in this architecture.

The work proposed in [38] uses a different blockchain model for IoT environments by eliminating the PoW consensus algorithm. In order to ensure privacy, the authors propose a lightweight privacy-preserving ring signature scheme. The privacy addressed at work resides in the user and device. The authors use an encryption method combined with a digital signature to ensure privacy. The authors do not present any gateway in their implementation due to the optimization of the consensus algorithm. The blockchain used is public. The data is stored off-chain in a cloud service. The main critique about this work is the use of smart contracts only for information verification of IoT devices.

Table 1 summarizes the information obtained in the analysis. The items showed in the table illustrate if the work addresses a specific aspect of the work. If the work does not specify any of the items, we used NE to denotate that. The table is divided into eight columns, as described below:

1. Reference;
2. Year;
3. Addresses user privacy;
4. Addresses user device;
5. Addresses environment device;
6. Blockchain type;
7. Blockchain used in implementation;
8. Communication gateway;
9. Storage used.

## 4. Proposed Architecture

**Figure 5:**
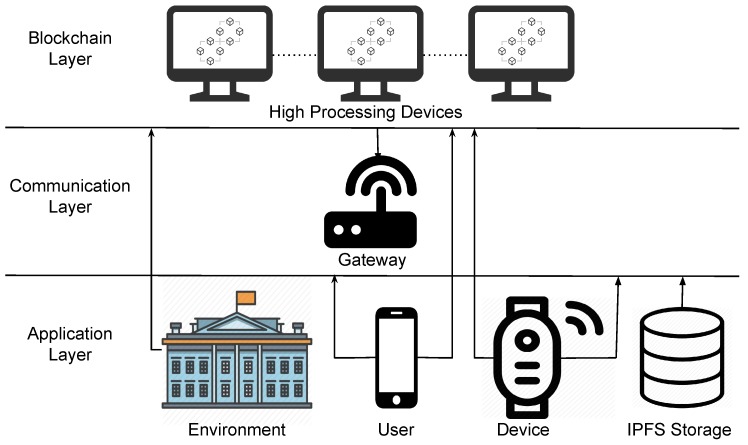
PRICHAIN Architecture.

Figure 5 illustrates the architecture overview. We divided the architecture into three layers called the blockchain layer, communication layer, and application layer. In the blockchain layer, smart contracts are stored, and transactions made in the environment are validated, in this layer, devices with considerable computational power are used to process blockchain requests. The communication layer is responsible for managing device-user communication. For this, a gateway is used considering that IoT devices do not have enough computational power to communicate directly with the blockchain. Finally, the application layer consists of the environment, user, and device, in which each will interact with each other through the decentralized mobile application developed. This layer also includes the IPFS storage service for data storage.

Considering the architecture illustrated in Figure 5 and the middleware described in Section 2, PRICHAIN is a decentralized implementation of UbiPri middleware. Using smart contracts combined with the communication gateway and IPFS storage service, PRICHAIN ensures the functionality of UbiPri modules. The use of different smart contracts, one for the environment, one for device and one for the user, favors the implementation of UbiPri in a decentralized way. The decentralized IPFS storage service and communication gateway assist in the implementation of the other middleware modules.

### 4.1. Smart Contracts

**Figure A1:**
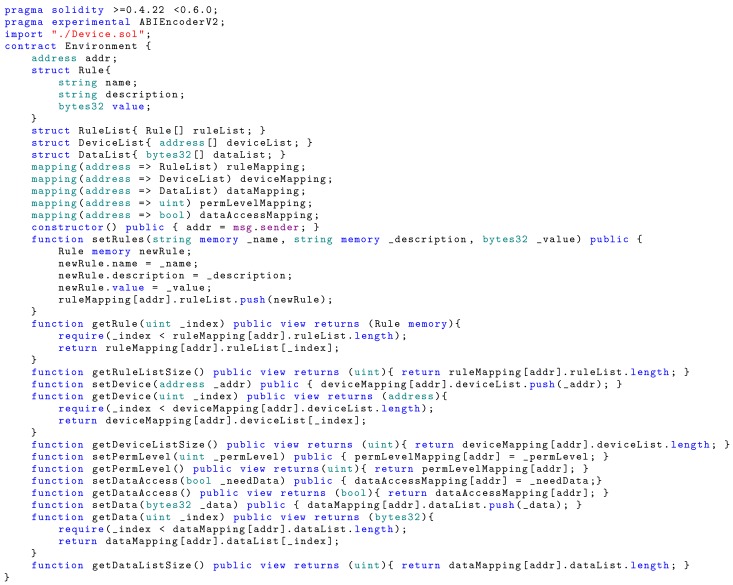
Environment Smart Contract.

**Figure A2:**
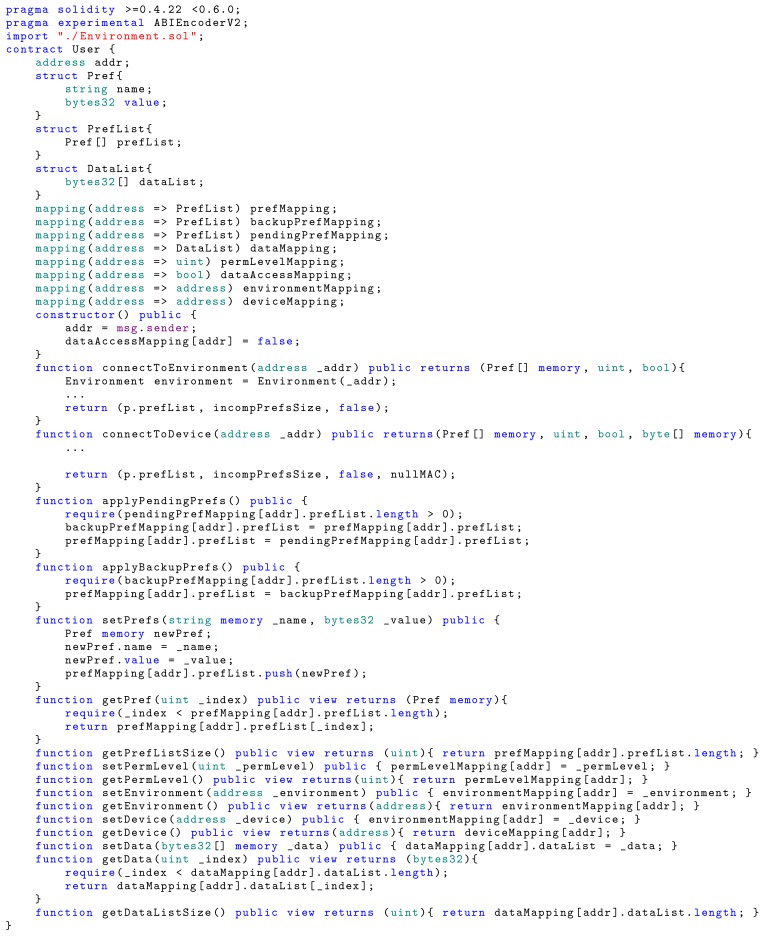
User Smart Contract.

**Figure A3:**
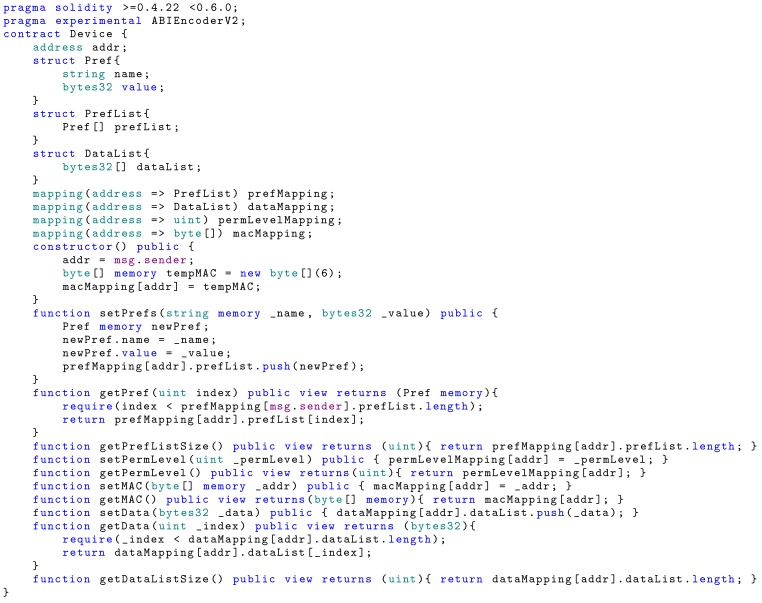
Device Smart Contract.

As mentioned previous, one of the UbiPri middleware assumptions refers to the concept that the environment denotes privacy rules. Therefore, we chose to use a smart contract specifically for the environment. The user has a separate smart contract with its individual privacy preferences. This  decision was made as the user may not agree with the environment rules. Finally, devices (sensors and actuators) also have smart contracts with their privacy preferences. Figure A1, Figure A2 and Figure A3 illustrate the contracts developed, the contracts were based on the proposed implementation in [39].

#### 4.1.1. Envinronment Smart Contract

The Environment contract has a rule structure based on an identification string, a rule description string, and a byte field of values. The user can also set a value for the permission level required to interact with the environment, and whether or not the environment will require access to user data history. The User contract has a structure of privacy options, also containing identification and value fields. The user can set the access level, such as whether or not they will allow access to their data history. A method called connectToEnvironment() requires the address of an Environment to verify that privacy, access level, and data usage options are compatible across contracts, returning incompatible items if any. The connectToDevice() method performs the same operations but based on a list of devices present in the environment, where the user can discover services. Pending operations framework store incompatible privacy options, which the user can apply if he/she agrees with them. Before each exchange, the old privacy values are stored in a backup structure so that the user can return them at any time. Finally, the Device contract also has a list of required privacy, permission to use user history, and required permission level. Also, the MAC address of the physical device is stored by the contract, which will be returned if the user can successfully connect to the device.

#### 4.1.2. User Smart Contract

The user can communicate with an environment by providing their address as an argument during a connectToEnvironment() operation. From this, the compatibility between user preferences and environment rules will be tested as well as the required permission level. In case of mismatch, Blockchain will return a list of frames including the name and value of missing or mismatched preferences, as well as the number of items in that list and a logical value indicating whether the operation was successful or not. In the case of compatibility, the environment contract will pair with the user contract in case of compatibility.

#### 4.1.3. Device Smart Contract

Being connected to the environment, a list of devices will be available in the contract and presented to the user. The user, in his contract, can enter as an argument the target device address within the connectToDevice() function. All preferences, as well as the required permission level, will also be tested by returning, in case of failure, the incompatible preference list, list size, valid value operation code, and null MAC address. If the communication is successful, the Android app redeems the corresponding MAC address of the device.

**Figure 6:**
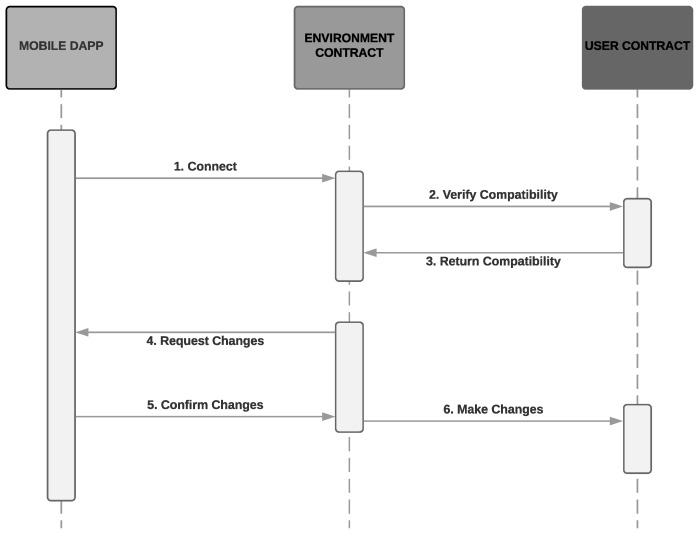
User-Environment communication.

Figure 6 illustrates the sequence diagram of communication between user and environment, described at the beginning of the section.

**Figure 7:**
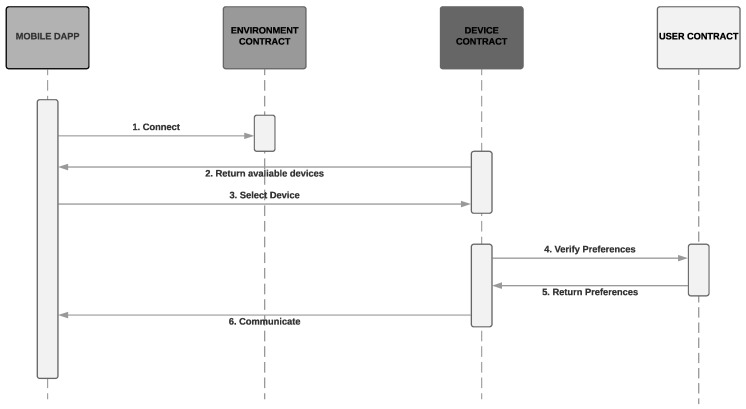
User-Device communication.

Figure 7 illustrates the sequence diagram of communication between user and device, described at the beginning of the section.

### 4.2. Communication Gateway

The gateway mediates communication between the user and the device. The communication protocol adopted was CoAP, using the library CoAPthon3, for the Python language. All operations performed are based on the resources available through the gateway. A device may perform a POST operation to provide a new service (or feature) that is identified by concatenating its MAC address with the type of service offered (e.g., CA: FE: CA: FE: CA: FE-LED).

Thus, a device can be configured to update a resource with the values of a sensor, or perform a periodic GET on a resource to obtain its value, as in the state of an LED changed by the user. When one of the points operates with PUT, the gateway reads the source, target MAC address, and communication data. This read information will be immediately sent to an IPFS node using ipfs-API, where it will remain unchanged. The user will be able to connect to a device resource via the MAC returned from the pairing function available in Smart Contracts, which concatenates with one of the discovered services names.

### 4.3. Mobile DApp

Dapps or Decentralized Application arising from Ethereum platform and their main objective is decentralization. For this, a dapp application must have the following principles:

- The application must be completely open source, it must operate autonomously and with no entity controlling most of its tokens.
- The application may adapt its protocol in response to proposed improvements and market feedback, but all changes must be decided by consensus of its users.
- Application data and operation records must be stored cryptographically in a decentralized public blockchain.
- Use a token that is required to access the app and any important contributions should be rewarded with the app tokens.
- The application must generate tokens according to a standard cryptographic algorithm that acts as proof that the nodes are contributing to the application.

As proof of concept and validation purposes of the proposed architecture, a smartphone application was developed using the Web3 library based on the React platform. The web app written in JavaScript and HTML5 provides the user interface for the Ethereum client, which in turn interacts with the blockchain.

**Figure 8:**
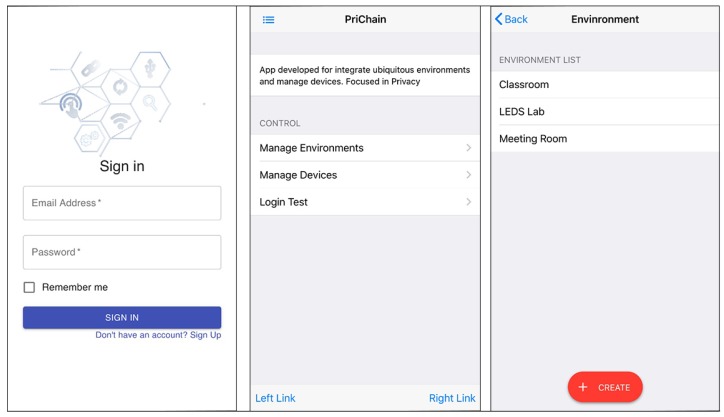
PriChain DApp.

The main purpose of this application is to interact with the three smart contracts (User, Environment and Device) mentioned above. Figure 8 represents the login screen for the application user to control the environment, the menu with available options and finally the list of available environments for user interaction.

## 5. Tests and Results

The hardware used was a 16-bit Arduino Uno R3 architecture operating at 16 MHz and 2 KB SRAM along with Ethernet Shield W5100. The sensors used were a 16-bit DHT11 resolution to measure temperature and humidity and an HC-SR04 ultrasonic sensor to check for the presence of people in the environment. A notebook with a 4-core Intel Core I5 8265U Dell notebook and eight threads operating at 1.6 GHz with 8gb DDR4 RAM and a 64-bit Kubuntu 18.04 operating system hosted the gateway and IPFS service. CoAP communication on Arduino was implemented through the SimpleCoAP library available on the Arduino IDE library manager.

The sketch used in Arduino occupied 19,998 bytes of the 32,256 bytes of available flash storage. It did not use EEPROM storage and 785 bytes of 2048 of available dynamic memory on the device representing 38% of the total. A local network simulated the environment. Therefore the latency of transmission by the network was disregarded.

The Ropsten TestNet was used as a private blockchain to test the interaction between and with contracts at no production cost. The average time to mine a block is 15 s, its standard gas price is 4 GWei, and the gas limit per block is 6,721,975.

### 5.1. Blockchain Performance

Table 2 shows the cost of implementing each of the three contracts in the network, considering a gas price of 20 GWei, which is the default for contracts due to the speed required in the transaction.

We note that the complexity of the logic of each contract contributes greatly to its cost. The User contract is the most complex and costly because of the functions that pair it with Environment and a Device, which instantiate other contracts, compare preference structures, store data, and access lists. On the other hand, Environment and Device contracts are logically simple and similar, which contributes to a relatively low cost. In Table 3, Table 4 and Table 5 some methods of each contract were chosen for further cost analysis based on their recurrence in normal use of the architecture.

As seen, the most costly functions are those that deal most with memory access, such as applyBackupPrefs() and applyPendingPrefs(), which deal with the user preference list. Although the connectToDevice() and connectToEnvironment() functions are logically more complex, computationally they are the least expensive.

In the Device contract, only one function has been tested, which is the most relevant to the architecture. This function only handles a six-position byte array and is therefore not as expensive.

Again, the most expensive functions presented, such as setRule() and setDevice(), are those that most interact with lists. On the other hand, functions that only change a single contract value, such as setDataAccess() and setPermLevel(), have a low cost.

### 5.2. Gateway Performance

Four distinct classes divide the tests about the implemented Gateway: Processing Time, Device Request Time, User Request Time, and IPFS Insertion Time. The operations performed and measured were those of GET and PUT, due to the consequence of their use in communication. The message size chosen was 4 bytes, as it is an average payload of all environments defined for this article. However, the IPFS node will receive the message along with the source MAC and request MAC target, totaling 38 bytes for its specific communication. The number of samples is 1, 10, and 100 shipments. In Table 6, we can see some of these results for the PUT method.

User request time was the longest among them due to internal network latency, machine-to-machine communication time, and gateway processing time. For the gateway, a PUT operation takes the most processing time because of the need to read the payload and update the CoAP resource. The device request time remained stable throughout the test. IPFS insertion, which counts the time of local node communication and message transfer, was the least expensive of the steps involved. Table 7 shows the same results for a GET operation, however without the IPFS insertion time, which is a characteristic of the PUT operation only.

### 5.3. Privacy

**Figure 9:**
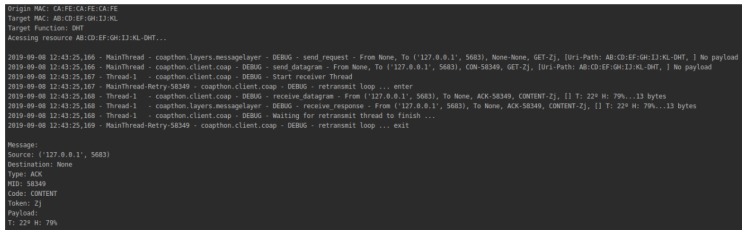
User communication with a device authorized by blockchain.

Figure 9 illustrates a user’s communication with the DHT11 sensor. User privacy preferences satisfy environment and device privacy preferences. Thus, the user can connect to the device and get the data related to it.

**Figure 10:**
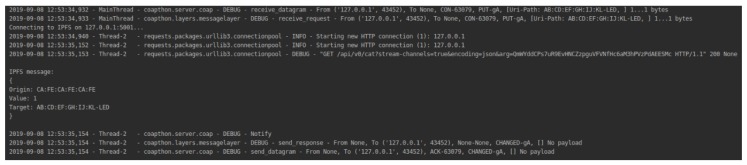
IPFS storage process.

In this situation, the user also chose to store the communication data. Figure 10 illustrates the gateway’s connection to the IPFS service.

**Figure 11:**
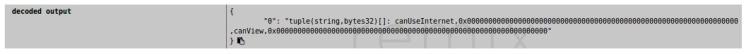
Blockchain denying the connection.

Figure 11 shows a user attempt to interact with the HC-SR04 sensor, but in this situation, the user’s privacy preferences do not match the device’s privacy preferences, so attempting to establish communication with the sensor is declined by blockchain.

### 5.4. Security

For security testing, communication with a local IPFS node has been established. According to its operation, recorded in a CoAP (PUT) resource is transferred from the IPFS API. The called function reads the message content and concatenates it with the MAC address of the source and target device. IPFS generates a hash about this message. Each hash generated corresponds to an address with immutable content within the network. Due to that, no transaction data will be improperly changed. The work aimed at abstracting the functioning of IPFS and, therefore, the address is not returned and saved in the user contract, but there is the possibility of an easy implementation of this mechanism.

## 6. Conclusions and Future Work

In this paper, we present a blockchain architecture for use in IoT environments. The UbiPri middleware, which the central objective is the privacy treatment in IoT environments, was used as a basis for the proposed architecture. We use a decentralized application to make the interaction between user, environment, and device. We can also ensure privacy at different levels by generating different smart contracts. Due to scalability issues, we chose to use a gateway for device-user communication. To ensure the integrity of stored data, we use the IPFS service, also ensuring data security.

With the tests developed, we realize that the architecture proposed in this work is feasible for implementation in different IoT environments. The results obtained concerning smart contracts show that the complexity of the contracts directly influences its cost. The User contract has the most functions, consequently being the most costly contract. Regarding the gateway, we realize that the results show that the implementation is scalable even with the latency generated by machine-to-machine communication and gateway processing time. In the privacy tests developed, we note that user-defined preferences are satisfied, however, when a user does not agree to change their privacy preferences to suit their environment, they cannot communicate with devices, smart contracts proprieties guarantees the privacy preferences. Regarding security, the integrity of the data stored in the IPFS service is guaranteed through a hash function. However, in this article, we aimed to abstract the IPFS service.

The results obtained with the development of this work contribute to other research that is being developed in the area of privacy. The use of the Ethereum blockchain has shown different costs for each contract. We can emphasize that they can be adapted to other types of blockchain. The design of the contracts was based on different state-of-the-art solutions. Therefore, this work also contributes to several areas of IoT that use smart contracts.

For future work, we suggest optimizing the contracts developed to reduce their cost. Regarding the gateway, we suggest the study of new technologies that can eliminate its implementation in the proposed architecture, making it completely decentralized.

## Figures and Tables

**Table 1:** Related Work.

Work	Year	User Privacy	Device Privacy	Environment Privacy	Blockchain Type	Blockchain Used	Gateway	Storage
[32]	2017	No	Yes	No	Consortium	Ethereum	No	Offchain
[30]	2017	Yes	Yes	No	Private	Ethereum	Yes	NE
[34]	2018	No	Yes	No	Private/Public	Ethereum	Yes	Offchain
[33]	2018	No	Yes	No	Private	Ethereum	Yes	Offchain
[35]	2019	No	Yes	No	NE	Ethereum	No	Offchain
[36]	2019	Yes	No	No	Public	IOTA	No	NE
[37]	2019	Yes	No	No	Private/Public	NE	No	NE
[38]	2019	Yes	Yes	No	Public	NE	No	Offchain
This Work	2019	Yes	Yes	Yes	Private	Ethereum	Yes	Offchain

**Table 2:** Implementation cost of each contract.

Contract	Transaction Fee (ETH)	Gas Used
User	0.06779326	3,389,663
Device	0.0277857	1,389,285
Environment	0.02598354	1,299,177

**Table 3:** User contract cost.

Function	Transaction Fee (ETH)	Gas Used
applyBackupPrefs	0.0004098	102,450
applyPendingPrefs	0.00040932	102,330
setPref	0.000341036	85 259
connectToDevice	0.000249916	62,479
connectToEnvironment	0.000193336	48,334

**Table 4:** Device contract cost.

Function	Transaction Fee (ETH)	Gas Used
setMAC	0.000192317	39,532

**Table 5:** Environment contract cost.

Function	Transaction Fee (ETH)	Gas Used
setRule	0.000370504	92,626
setDevice	0.000256304	64,076
setDataAccess	0.000170144	42,536
setPermLevel	0.000168932	42,233

**Table 6:** PUT Results.

Samples	Processing Time (ms)	User Request (ms)	Device Request (ms)	IPFS Insertion (ms)
1	8.945	24.615	1.497	0.121
10	78.592	236.142	14.96	1.39
100	1071.41	2669.34	149.6	26.14

**Table 7:** GET Results.

Samples	Processing Time (ms)	User Request (ms)	Device Request (ms)
1	Negligible	13.045	1.440
10	0.004	130.79	14.37
100	0.055	1293.0	160.0

## Appendix A

Figure A1 illustrates the contract developed to manage the privacy preferences of the environment. This contract is responsible for define the environment rules and interact with the user. The algorithm is described in detail in Section 4.1.1.

## Appendix B

Figure A2 illustrates the contract developed to manage the privacy preferences of the user. This contract is responsible to set the user privacy preferences and interact with the environment. The algorithm is described in detail in Section 4.1.2.

## Appendix C

Figure A3 illustrates the contract developed to manage the privacy preferences of the devices. This contract is responsible to set the device privacy preferences and interact with the user and environment. The algorithm is described in detail in Section 4.1.3.
